# Exploring the relationship between shared identity and interoperability: a mixed methods analysis of discussion-based multi-agency emergency response exercises

**DOI:** 10.1080/10439463.2024.2374834

**Published:** 2024-07-09

**Authors:** Louise Davidson, Holly Carter, John Drury, Richard Amlôt, S. Alexander Haslam

**Affiliations:** a School of Psychology, University of Sussex, Brighton, UK; b Behavioural Science and Insights Unit, UK Health Security Agency, Porton Down, UK; c School of Psychology, University of Queensland, St Lucia, Queensland, Australia

**Keywords:** Shared identity, interoperability, major incident management, multi-agency emergency response

## Abstract

Previous research has shown ongoing difficulties between Police, Fire and Rescue, and Ambulance Service responders during multi-agency emergencies. Recently, researchers have used the Social Identity Approach to gain insight into these challenges, offering a psychological framework for understanding relations within and between response organisations. This study builds upon previous work by engaging responders from the emergency services in six discussion-based exercises. By analysing participants’ identity levels and their perceptions of joint working performance, we found a positive association between shared identity and interoperability. Analysis of the discussion transcripts highlighted areas where joint working faced obstacles, such as the use of organisation-specific terminology. Furthermore focus group discussions after the exercise revealed key factors linking shared identity to effective multi-agency response, including increased motivation to collaborate and increased trust and respect. This research deepens our understanding of multi-agency working from a social identity perspective, highlighting the importance of shared identity in enhancing joint efforts. Practical implications are addressed.

## Introduction

The UK emergency services’ multi-agency response faces recurring challenges across incidents, as shown in inquiries into events including the 7/7 bombings (Hallett 2011), Hillsborough Stadium Disaster (Taylor 1990), and the Manchester Arena attack (Saunders 2022a). For example, the Manchester Arena inquiry noted ‘significant failures’ in communication and decision-making among Police, Fire and Rescue (FRS), and Ambulance Service responders resulting in conflicting information and no clear understanding of the incident (Saunders 2022a, 2022b). Similar issues have been reported in other countries too, such as the 2001 World Trade Centre Attacks (Kean and Hamilton 2004) and the Haiti earthquake (Patrick 2011). These persistent challenges highlight the need to better understand and address multi-agency response issues.

Recent research uses the Social Identity Approach to understand interoperability challenges (Davidson *et al*. 2022, 2023). This study applies the same approach to explore how responders’ shared identity affects their teamwork in a discussion-based exercise. To explain the rationale for why we chose this approach in the current study, we will first introduce interoperability and then explain how social identity processes relate to these challenges.

### Interoperability

Interoperability, the ability of responders from different organisations to work together coherently (Joint Emergency Services Interoperability Programme [JESIP] 2013), has been a key focus in UK emergency management for over a decade (Pollock 2013). JESIP, introduced in 2012, aimed to address these challenges by introducing standardised joint working principles (e.g. clear communication, see Table 1). However, interoperability issues persist (Pollock 2021). The 2017 Manchester Arena attack inquiry criticised emergency services for not embedding JESIP principles into their practices (Saunders 2022a). This is in line with findings from Pollock (2013) who suggested that for interoperability to be effective, it needs to be embedded within the organisational culture of the emergency services.

**Table 1.** The five JESIP principles for joint working, from JESIP (2021).

Principle	Definition
Co-locate	Co-locate with other responders as soon as practicably possible at a single, safe, and easily identified location
Communicate	Communicate using language which is clear, and free from technical jargon and abbreviations
Co-ordinate	Co-ordinate by agreeing the lead organisation. Identify priorities, resources, capabilities, and limitations for an effective response, including the timing of further meetings
Jointly understand risk	Jointly understand risk by sharing information about the likelihood and potential impact of threats and hazards, to agree appropriate control measures
Shared situational awareness	Establish shared situational awareness by using M/ETHANE (a structured mnemonic model for responders to collate and pass on information, including whether a major incident has been declared [M] and the exact location of an incident [E]), and the Joint Decision Model (a model to help responders make decisions together in a response considering the need for immediate action to save lives and reduce harm, including gather information and intelligence, and identify options and contingencies)

Organisational culture comprises the roles, norms, and values that create shared meaning for its members (Ellemers 2003, Haslam 2004). A recent review emphasised that embedding interoperability into organisational culture is crucial (Power *et al*. 2024). Davidson *et al*. (2023) argue that a shared identity, where members feel, and feel to be seen as, part of the group is essential for fostering an interoperable culture. This sense of belonging enhances commitment to the organisational culture (Haslam 2004). The role of shared identity in interoperability is further discussed below.

### Shared identity processes and interoperability

The Social Identity Approach (Tajfel and Turner 1979, Turner *et al*. 1987) provides insight into how people from different groups work together. Accordingly, a personal identity describes a person in contrast to others (e.g. ‘I’, ‘me’), whereas a social identity psychologically connects people through a sense of ‘we’ and ‘us’ (i.e. people who view each other as members of a common group; Neville *et al*. 2022). A sense of shared identity (i.e. a shared sense of ‘us-ness’) within a group can provide group members with shared definitions of situations and common norms for behaving in those situations (Reicher *et al*. 2010), foster trust and respect among group members (Turner *et al*. 1987, Haslam *et al*. 2012), and facilitate coordination and cooperation between group members (Haslam *et al*. 2009, 2022).

A key complexity present in the multi-agency response that can make coordination and cooperation between responders difficult is that they require usually independent teams of Police, FRS, and Ambulance responders to work together collaboratively to achieve a shared goal (Bharosa *et al*. 2010). In other words, it involves the formation of a multiteam system (MTS; Marks *et al*. 2001, Mathieu *et al*. 2001). Importantly, not only are these teams within the MTS working towards a shared goal, they often also have differing and potentially competing sub-goals that they must achieve in the pursuit of this shared goal (Power and Alison 2017). Crucially, the sub-teams within the MTS must work together to manage these sub-goals, as well as differing command structures, language, and procedures to provide an effective joint response (Brown *et al*. 2021).

An important consideration for MTS’s is that people can have multiple social identities which can become salient in different contexts (Connaughton *et al*. 2011, Millward and Haslam 2013, Luciano *et al*. 2018). For example, in a multi-agency response team, responders can identify both with their sub-team (Police, FRS, Ambulance) and their superordinate team (emergency responders). Research on military teams in the Netherlands showed that salient sub-team identities were associated with less trust and more conflict between sub-teams (Wijnmaalen *et al*. 2019). Additionally, a study using students in a simulated firefighting scenario showed that identification with the emergency response MTS improved MTS performance, emphasising the role of shared identity in MTS performance (Cuijpers *et al*. 2016).

Recent research has sought to expand on this by applying the Social Identity Approach to Police, FRS, and Ambulance responders joint working. Davidson *et al*. (2022, 2023) conducted two interview studies with responders involved in the multi-agency COVID-19 response. They found evidence of shared identity among responders which was enhanced by a shared purpose, shared difficult experiences, and leaders strategically reinforcing this sense of identity.

Davidson *et al*. (2022, 2023) provide useful insight into the presence of a shared identity between responders in these multi-agency teams. However, this research does not explore an association between shared identity and interoperability within these multi-agency teams. Previous research shows that shared identity improves MTS performance (Cuijpers *et al*. 2016, Wijnmaalen *et al*. 2019, Mell *et al*. 2020). While Cuijpers *et al*. (2016) and Wijnmaalen *et al*. (2019) suggest a link between shared identity and MTS performance, this (to our knowledge) has not been studied with real emergency responders during multi-agency emergency response. Our study aims to fill this gap by using discussion-based exercises with Police, FRS, and Ambulance Service responders to explore any associations between shared identity and interoperability.

### The role of exercises in emergency response research

Exercises are essential for assessing emergency response capabilities (Borell and Eriksson 2013) and include live exercises (e.g. Skryabina *et al*. 2017), immersive simulations (e.g. Brown *et al*. 2020), and discussion-based exercises (e.g. Borell and Eriksson 2013). Whilst live exercises and simulations offer higher fidelity than discussion-based exercises (Alison *et al*. 2013), we chose discussion-based exercises for this study due to epistemological and logistical reasons. These exercises build on previous interview-based research and involve discussing a plan or scenario without needing real-time properties (Borell and Eriksson 2013, Skryabina *et al*. 2017). When developing discussion-based exercises, Borell and Eriksson (2013) recommend incorporating variation into the exercise to provide different response experiences, using scenarios to provide this variation within and/or between scenarios, and providing interaction opportunities between participants to generate insights into different perspectives and opportunities for collaboration. We incorporated these three recommendations in the current study, as discussed below.

### The current study

Given the role of shared identity in MTS’s and evidence of its presence in multi-agency response teams, this study used discussion-based exercises to explore the link between responders’ shared identity and their ability to work together. Specifically, this research aimed to answer the following research questions:

RQ1. Did participants experience a shared sense of identity with each other during the exercises?

RQ2. Did participants think they worked effectively together during the exercises?

RQ3. Was participants’ shared identity linked to their perceptions of how effectively they worked together during the exercises?

RQ4. How did participants work together during the exercises?

RQ5. How was shared identity linked to improved joint working?

## Method

### Design

Participants took part in two discussion-based exercises in groups of three to six (six groups in total). Using a within-subjects mixed-methods design, both quantitative and qualitative data were collected. The design had two factors: time (with three levels: baseline, post-exercise 1, and post-exercise 2) and exercise (with two levels: Exercise 1 and Exercise 2). Quantitative data came from questionnaires at each time-point, while qualitative data came from exercise transcripts and post-exercise focus groups.

### Participants

Twenty-four current or former operational commanders from Police (*N* = 7), FRS (*N* = 15), and Ambulance (*N* = 2) Services from six regions across the UK participated. They were recruited through emergency service contacts and word of mouth. Participants were grouped regionally to minimise any between-region differences that may interfere with responders’ ability to work together. Due to availability and operational commitments, group sizes and service representation varied, but each group included representatives from at least two emergency services. Participants averaged 20 years of service (range: 8–30 years). Except for Groups 2, 4, and 6, where one participant lacked a pre-existing relationship, all participants knew at least one other group member before the exercise. Most reported participating in multi-agency training every 6–12 months and were ‘very’ or ‘extremely’ familiar with JESIP. See Table 2 for participant group details by organisation and gender.

**Table 2.** Participant information.

Group	Organisation	Gender
1	Police: 2 (33.3%) FRS: 3 (50%) Ambulance: 1 (16.7%)	Male: 5 (83.3%) Female: 1 (16.7%)
2	Police: 1 (33.3%) FRS: 2 (66.7%) Ambulance: 0 (0%)	Male: 3 (100%) Female: 0 (0%)
3	Police: 1 (25%) FRS: 3 (75%) Ambulance: 0 (0%)	Male: 4 (100%) Female: 0 (0%)
4	Police: 0 (0%) FRS: 2 (66.7%) Ambulance: 1 (33.3%)	Male: 3 (100%) Female: 0 (0%)
5	Police: 1 (33.3%) FRS: 2 (66.77%) Ambulance: 0 (0%)	Male: 3 (100%) Female: 0 (0%)
6	Police: 2 (40%) FRS: 3 (60%) Ambulance: 0 (0%)	Male: 5 (100%) Female: 0 (0%)

### Materials

#### Exercises

Following Borell and Eriksson’s (2013) advice, the exercises featured two distinct and evolving scenarios: a flood at a primary school where several children and staff were trapped inside the school building and needed rescuing (Exercise 1); and a shooting at a restaurant where several members of the public were injured and there was conflicting information surrounding the location of the shooter (Exercise 2). Participants received updates on scenario developments during the exercise. Scenarios were developed in consultation with subject matter experts from the emergency services to ensure the situations and challenges presented were representative of what might occur in a real incident. All groups completed both exercises, with the order counterbalanced across groups (see Supplementary Materials 1 for full scenarios).

#### Pre-exercise questionnaire

The pre-exercise questionnaire included two items on participants’ identity: ‘I feel a bond with the other responders taking part in this exercise’ and ‘I think that the other responders taking part in this exercise feel a bond with each other’. Adapted from Leach *et al*. (2008) items for solidarity and in-group identification, these items avoided the term ‘identity’ to prevent priming participants. Items showed good internal reliability (*r*_sb_* *>* *0.90). See Supplementary Materials 2 for the full pre-exercise questionnaire.

#### Post-exercise questionnaire

The post-exercise questionnaire contained the same scale as the pre-exercise questionnaire and showed good internal reliability at both time-points (*r*_sb_’s* *>* *0.90). It also included a scale on perceived joint working performance (e.g. ‘I communicated effectively with other responders taking part in this exercise’, 5 items). Items were adapted from the JESIP Assurance Framework (2017), the standard training for interoperability in the UK at the time this research was conducted. These items showed good internal reliability (*α *>* *0.90). See Supplementary Materials 3 for the full post-exercise questionnaires.

#### Focus group topic guide

For consistency across measures, the focus group guide covered themes similar to those in the post-exercise questionnaires. Themes related to participants’ shared identity and their perceived joint working performance. See Supplementary Materials 4 for the full focus group topic guide.

### Procedure

Ethical approval was obtained fromUK Health Security Agency Research and Governance Group, approval number: R&D 477. Because of constraints on in-person meetings due to the COVID-19 pandemic, exercises were conducted virtually via Microsoft Teams. The first author ran all exercises. All groups, except Group 3, logged in separately. Group 3 shared a computer in the same room.

After logging into Microsoft Teams participants received a link to the online survey. Participants read the information sheet and were able to ask the researcher any questions before signing an electronic consent form. Participants then completed the pre-exercise questionnaire.

After finishing the pre-exercise questionnaire, participants completed Exercise 1. The researcher provided the initial exercise information via Microsoft Teams chat. Participants had 15 min to discuss their response, with subsequent updates every (approximately) 12 min. Discussions took place verbally and each exercise lasted 1 h. Following Exercise 1, participants completed the first post-exercise questionnaire. Participants then completed Exercise 2 following the same timelines as Exercise 1, before completing the second post-exercise questionnaire. Finally, participants took part in a 30-min semi-structured focus group led by the researcher which lasted approximately 30 min (range 25–36 min). Participants received a certificate of participation by email. Exercises were recorded and transcribed by the first author.

### Analysis

#### Questionnaire data

Questionnaire data were analysed using SPSS 28.0.1. Non-parametric tests were used due to violated parametric assumptions and were conducted as two-tailed tests. One-sample Wilcoxon Signed Rank tests compared identity and joint working performance scores to the midpoint value of 4 on the Likert scale to assess shared identity (RQ1) and joint working performance (RQ2). A Friedman’s test explored changes in identity scores and a Repeated-Sample Wilcoxon Signed Rank test compared joint working performance between Exercise 1 and 2. Spearman’s correlations examined relationships between identity scores and joint working performance (RQ3). We conducted effect-size sensitivity analyses using G*Power (Faul *et al*. 2007) for each statistical test to ensure adequate power to detect effects (Giner-Sorolla *et al*. 2022).

#### Discussion transcript data

Data was analysed using the framework approach (Pope *et al*. 2000). A thematic framework was identified based on the joint working principles laid out in the Joint Doctrine (JESIP 2021): communicate; coordinate; co-locate; joint understanding of risk; and shared situational awareness (see Table 3 for coding framework). Relevant passages within the data were coded into one or more of the themes outlined in the coding framework. The first author coded the dataset and met with the research team to discuss the analysis.

**Table 3.** Coding framework, with illustrative quotes from data, for the discussion transcript data analysis, adapted from JESIP (2017) Exercise Assurance Framework.

Code	Description	Illustrative quote
Co-locate	Whether responders discussed co-locating with each other: Did responders discuss meeting face to face? Was a forward command point established? Did commanders identify timely on-scene briefings?	‘I think at this point we would slowly be starting to get into our battle rhythm, and we’d be identifying you’ve got your world to look after, I’ve got my world to look after, we probably set an agreed, let’s come down every 10 minutes, every 15 minutes […] although we won’t necessarily be living in each other’s pockets, but we’d certainly have that let’s meet up at appropriate points, so that communication thing would be would be switched on’ (Police, Group 2)
Communicate	The language used by responders and how it was shared between responders: was common terminology used? Was relevant information shared across services? Was M/ETHANE used to pass information?	FRS: ‘you know, a quick and dirty silver meeting to erm just confirm those things […]’ Police: ‘Whilst I’m really comfortable that silver meetings are really important and I think they are, erm sometimes […] can be done outside of a silver meeting […]’ FRS: ‘Yeah, I think probably different understanding of of a silver meeting there. I’m quite comfortable comfortable to have that like a quick discussion over the bonnet and decide those things’ (Group 6)
Co-ordinate	How responders discussed co-ordinating their actions: Were joint decisions on priorities made? Were actions joined up between responders? Was duplication of effort negated? Was there an understanding of the capability, capacity, and limitations of each other’s assets?	‘So the fact that the media have arrived and cordons and all the other bi, is our problem to a certain point, but it also isn’t. I’d be expecting them to remain outside that cordon and I’m not getting involved’ (FRS, Group 2)
Jointly understand risk	Whether responders shared an understanding of the risk of the incident: Were threats and hazards identified, understood, and treated differently by each emergency service? Were limitations of people and equipment identified, understood, and treated different by each service? Were capabilities of people, equipment identified, understood, and treated different by each service?	‘From a police officer that is not swift water trained or anything, when I read three foot I’m mindful of not understanding what the risks are […] and I think a very brief conversation […] so an understanding of that risk, that joint understanding of risk of why that is a major incident and why we can’t just walk straight in […] would be quite important to understand’ (Police, Group 6)
Shared situational awareness	Whether responders had a common understanding of: What had happened, what was changing throughout, the consequences of the events, priorities and objectives, each service’s role in resolving the incident	‘We need to get them. Er so further flash flooding, what’s that gonna look like, but also now I’m thinking about, from our point of, I’m thinking about officer safety welfare. What are the implications for emergency service personnel deployed […] are there any other safe systems of work that needs to be brought in, and also to facilitate that? But again, the response is still yours. We’re still, from a policing point of view, we’re still securing the area and supporting you in delivering it’ (Police, Group 3)

#### Focus group data

Data was analysed using semi-deductive thematic analysis (Braun and Clarke 2006). This analysis approach was chosen because, whilst there were no predetermined themes, the Social Identity Approach provided a general reference point to guide the analysis. The first author reviewed the transcripts, noting initial codes related to RQ5 and discussed potential themes with the research team. Transcripts were re-reviewed and relevant extracts were coded to these themes. The research team then finalised and defined the themes, which included shared goals, shared frame of reference, motivation to work with each other, confidence, and increased trust and respect (see Table 4).

**Table 4.** Thematic framework and illustrative quotes.

Theme	Description	Illustrative quote
**1. Development of a shared identity**		
1.1 Shared goals	Any discussions about how any goals shared between responders from different response organisations influenced their ability to work together as a team or influenced the development of a bond or identity	‘I think ultimately […] that professional relationship, I would say we’re all pulling in the same direction. I think we we know that we want to have the best, most efficient way of resolving the incident erm that’s the nature of us. I think working in the public sector and so everyone’s got the same same goal’ (FRS, Group 5)
1.2 Shared frame of reference	Any discussions around how shared principles (e.g. JESIP) or shared intrinsic values between responders from the different response organisations influenced their ability to work together as a team or influenced the development of a bond or identity	‘I think it’s that that common language […]. I think [Name of Ambulance responder] used about OPS discretion and confirmed that we use that as well er so we we did establish that that common playing field that ground that we all can work two and for so. Dare I say it, all the emergency service people are of a similar mindset, we’re there to to make a difference and to support each other and help out. So er you know, I think that’s really important that we are can do, will do, type of people rather than come up with problems, that we’ll look for solutions’ (FRS, Group 4)
**2. How shared identity and joint working are linked**		
2.1 Motivation to work with each other	Any discussions around how a shared bond between responders, or a shared sense of identity, increased participants motivation to work together, for example responders wanting to work with each other and share information in a way that is accessible to other responders	‘That’s your point of view, well, how can I put across my point of view and my understanding to you know In your terms or something’s gonna make a lot more sense for you erm so that’s the kind of the relationship that builds up that you sort See what the other person wants and needs and their way of thinking and trying to build into that and everyone gets into the middle’ (Police, Group 5)
2.2 Confidence	Any discussions around how a sense of a bond or identity, or how the relationships between responders, increased their confidence both in their own abilities, but also in each other’s abilities	‘The more we do it, the more comfortable we get about challenging each other as well, if we think something is not right […] if we think something is not right, then freedom to speak up, and just to challenge and maybe understand the risk or something that that I haven’t understood as a commander, is is easier to to approach and ask about and equally you know for me to be challenged’ (Ambulance, Group 1)
2.3 Increased trust and respect	Any discussions around how a sense of a bond or identity, or how the relationships between responders, increased their trust and respect in each other	‘As you find out who people are you you know and understand that wealth of knowledge that they can bring which automatically gives you a certain amount of respect which then automatically makes you want to work together. […] the more you do as a cadre of people, the more you perform better on the incident ground for me because you already have that relationship. There’s nothing better than turning up at a job and seeing a familiar face from another service, seeing that face you know you can rely on and I guess as the scenarios build, I guess in the first scenario, yeah, I respect his job. OK so after the first scenario he knows what he’s talking about, I’m gonna listen to him, he’s got this opinion, and that’s the that’s the relationship building which then, I can’t remember the exact word in your question, that that brings you to that kind of joint working together and JESIP-ness’ (FRS, Group 2)

## Results

This section is organised by the research questions. First, questionnaire data addresses RQs 1–3. Second, discussion transcripts address RQ4. Finally, focus group data address RQ5.

### Questionnaire data

This section is separated by RQs 1–3. Sensitivity analysis indicated sufficient power for RQs 1–2, but partial power for RQ3 (see Supplementary Materials 5).

#### RQ1. Did participants experience a sense of shared identity with each other during the exercises?

Participants’ identity scores were significantly higher than the midpoint (4) on the Likert scale at each time-point: baseline (*Mdn *= 5.75), *z *= 3.78, *p *<* *.01, *r *= 0.77; Time-Point 1 (*Mdn *= 6.00), *z *= 4.16, *p *<* *.01, *r *= 0.85; Time-Point 2 (*Mdn *= 6.00), *z *= 4.11, *p *<* *.01, *r *= 0.84. This suggests participants experienced a sense of shared identity both before and during the exercise.

Participants identity scores changed significantly over time, *χ*^2^ (2) = 13.20, *p *<* *.01. Post hoc analysis with Bonferroni-corrected (*p *<* *.017) Wilcoxon signed-ranks showed significant differences between baseline and Time-Point 1, *T *= 3.08, *p *<* *.01, and baseline and Time-Point 2, *T *= 2.89, *p *<* *.01. However, scores did not differ significantly between Time-Point 1 and Time-Point 2, *T *= .14, *p *= .89. This suggests that participants’ sense of shared identity increased after beginning the exercise, although no further increases occurred between Exercises 1 and 2.

#### RQ2. Did participants work effectively together?

Participants’ joint working scores were significantly higher than the midpoint (4) on the Likert scale at each time-point: Time-Point 1 (*Mdn *= 6.00), *z *= 4.23, *p *<* *.01, *r *= 0.86; Time-Point 2 (*Mdn *= 6.00), *z *= 4.24, *p *<* *.01, *r *= 0.87. This suggests that participants perceived that they worked well together during the exercise.

Participants’ joint working scores did not significantly differ between Time-Point 1 and Time-Point 2, *z *= 1.79, *p *= .07, *r *= 0.37. This suggests no improvement in perceived joint working in Exercise 2 compared to Exercise 1.

#### RQ3. Was participants’ sense of shared identity linked to improved joint working?

Participants familiarity levels with JESIP before taking part in the study was significantly related to their perceived joint working performance both during Exercise 1, *r*_s_ = 0.75, 95% BCa CI [0.599, 0.846], *p *<* *.01, and Exercise 2, *r*_s_ = 0.55, 95% BCa CI [0.195, 0.777], *p *<* *.01.

Participants’ identity levels after Exercise 1 was significantly related to their perceived joint working performance during Exercise 1, *r*_s_ = 0.47, 95% BCa CI [0.084, 0.745], *p *<* *.05, and Exercise 2, *r*_s_ = 0.75, 95% BCa CI [0.599, 0.846], *p *<* *.01, but not during Exercise 2, *r*_s_ = 0.27, 95% BCa CI [−0.168, 0.673], *p *= .19.

Participants’ identity levels after Exercise 2 were significantly related to their perceived joint working performance during both Exercise 1, *r*_s_ = 0.46, 95% BCa CI [0.059, 0.770], *p *<* *.05, and Exercise 2, *r*_s_ = 0.52, 95% BCa CI [0.142, 0.796], *p *<* *.01.

There was no significant relationship between participants’ identity levels before the study and their perceived joint working performance both during Exercise 1, *r*_s_ = 0.18, 95% BCa CI [−0.234, 0.561], *p *= .40 and Exercise 2, *r*_s_ = −0.12, 95% BCa CI [−0.503, 0.245], *p *= .58.

### Discussion transcript data

This section is presented in relation to RQ4: how participants worked together during the exercise. Themes are presented in relation to the five principles for joint working principles, as per JESIP (2021): co-locate, communicate, coordinate, jointly understand risk, and shared situational awareness.

#### Co-locate

Although physical co-location was not possible due to the study’s nature, all groups discussed co-locating during the exercises. However, the depth of this discussion varied across groups. Some groups briefly mentioned co-location at the response’s outset (e.g. Group 4), whilst other groups discussed in more detail the importance of co-location and why they would do it. For example, in the flooding scenario, Group 1 emphasised co-location to share information and assess rescue urgency. Additionally, Group 6 explicitly mentioned being in the same location during the flooding exercise, while FRS responders requested Police to secure a cordon for safe equipment delivery.

Yet, despite seemingly clear communication regarding a shared meeting point in the flooding exercise, confusion arose in some groups during the shooting exercise. For example, FRS participants in Groups 5 and 6 were uncertain if they were attending a shared meeting point with the Police and Ambulance or an FRS-designated one. They stressed the necessity of meeting with Police in person to discuss important response aspects and identify safe areas of work.

#### Communicate

Five groups discussed using common frameworks for information sharing (e.g. M/ETHANE, JESIP 2021), though sometimes this information had to be requested. For example, in the shooting scenario, Group 6 initially discussed using M/ETHANE, but the conversation shifted towards intelligence gathering instead of following the framework. Major incident declaration, the framework’s first stage, was not addressed until 15 min in when an FRS participant requested this from the Police. Once declared, the conversation focused on practical actions rather than speculations. Similarly, in Group 2 an FRS participant mentioned the framework early, but it was not discussed again until 17 min in when the participant discussed formulating a M/ETHANE response.

Furthermore, whilst participants from different services used similar terminology, it is uncertain if they shared a common understanding of its meaning. For example, during the shooting scenario in Group 1, both Police and FRS participants used the term ‘clear’ when discussing scene safety and how close to the scene FRS participants were able to be, leading to apparent confusion between the Police and FRS. The FRS participant intended to deploy crews once the scene was clear, but Police disagreed stating FRS would not be allowed near the scene. To address potential misunderstandings, the Ambulance participant sought clarification from Police on their use of the word ‘clear’. Similarly, in Group 6 there was confusion over ‘Silver meeting’, requiring clarification between Police and FRS participants with differing understandings.

#### Coordinate

All groups agreed that FRS would lead the flooding scenario and Police would lead the shooting scenario. Additionally, when declaring a major incident, most participants supported other service’s needs, even if it was not a major incident for their organisation.

Furthermore, participants in some groups actively shared their capabilities and requested assistance from other services to support a coordinated response. For example, in the flooding scenario, the Ambulance participant in Group 1 clarified their organisation’s limitations, stating they do not operate in water. Also, FRS participants in Group 2 asked Police for support, such as setting up a cordon for safe evacuation from the school. Similarly, in the shooting scenario, FRS, and Ambulance participants in Group 1 sought information from Police on safe work areas.

However, sometimes conversations seemed insular, reflecting siloed or hierarchical thinking. Participants expected others to perform a certain task without confirming this with them. For example, in Group 5, a Police participant mentioned setting up a joint meeting point, assuming Ambulance responders would not, but they did not mention discussing this with them, risking duplicated action. Additionally, some participants dismissed issues outside their priorities. For example, in Group 6’s flooding scenario, a Police participant shifted responsibility to FRS without discussing stepping down the multi-agency response. Similarly, in Group 2’s shooting scenario, an FRS participant considered media arrival a Police concern, neglecting collective responsibility.

#### Jointly understand risk

All groups agreed that the leading organisation in the response would also lead the joint risk assessment. Despite one service leading, participants from the other services still asked questions to facilitate a shared risk understanding. For example, in the shooting scenario, FRS and Ambulance participants questioned Police participants about safe work areas, protective equipment, and known or potential risks.

However, in the flooding scenario, risks appeared less clear, causing confusion among participants around safe response actions. For example, in Group 6 Police participants were confused about why three feet of water posed a risk and why they could not wade through the water to rescue the trapped people. They emphasised the importance of FRS’s communication of this risk to prevent Police from ignoring safety procedures and entering the water.

#### Shared situational awareness

In several discussions across all groups and scenarios, participants emphasised the need for information gathering before making operational decisions. This was a key focus at the beginning of the scenarios when many details about the incident were unknown. For example, in Group 6’s shooting scenario an FRS participant explained they would not commit crews to the scene until more information was available. Hence, their immediate focus was clarifying the situation by communicating with lead responders from other services. However, this early need for intelligence seemed to hinder explicit decisions being made, specifically in the shooting scenario where more uncertainties were present.

### Focus groups

This section is presented in relation to RQ5, exploring how shared identity is linked to joint working. It is separated by two themes: The development of shared identity (shared goals, shared principles) and effect of shared identity on joint working (motivation to work with each other, confidence, and increased trust and respect). Themes are presented alongside relevant extracts from the focus group transcripts.

#### Development of a shared identity

This section is separated into two themes: shared goals and shared frame of reference.

*Shared goals.* In most groups, participants said they were trying to achieve the same goal, regardless of their organisation. Some specified what the goal was (e.g. ‘resolution of the incident’, ‘save life’), whilst others more broadly stated they were ‘all here for the same thing’ or their ‘goal is fundamentally the same’.

Participants noted shared goals provided a shared purpose in the response. One Ambulance participant in Group 1 highlighted how shared goals ensured participants were on the ‘same page’. Similarly, a Police participant in Group 2 emphasised shared goals facilitated a shared mindset creating a sense of being on the ‘same team’ despite organisational differences.

An FRS participant in Group 6 noted that despite different organisations’ approaches to achieving a shared goal, just having one can build a bond between them. Additionally, a Police participant in Group 5 acknowledged overarching goals, though priorities may differ. However, working together and recognising shared goals, as well as different priorities, improved working relationships. This participant also highlighted awareness of other organisations’ need beyond a singular (e.g. ‘police way’) approach.

*Shared frame of reference.* All groups acknowledged that JESIP provided a shared frame of reference, aiding cohesion during the exercise because they had a common guide for how to approach the scenarios and what to discuss. A Police participant in Group 3 said that this shared frame of reference helped to create a bond within their group. An Ambulance participant in Group 1 found reassurance in using JESIP principles to help guide their discussions and decision-making because it highlighted that they were ‘all on the same page’, emphasising shared understanding over organisation-specific procedures. This recognition of shared procedures fostered unity despite organisational differences:

I think [recognising similarities] helps massively […] ‘cause everyone can see everyone’s doing the best they can […] we realise we’ve all got the same kind of stresses and strains in our jobs and that kind of unifies people almost into that blitz spirit where everyone is just doing the best they can and we find we can do better with each other than we can in isolation […] out there or in here on this exercise. (Police, Group 2)

In addition to shared principles, participants highlighted psychological similarities, such as reasons for joining their organisations, and intrinsic motivations for being an emergency responder, as contributing to a shared frame of reference. Participants said this perception aided in viewing each other as part of the same group. For example, a participant in Group 4 said despite varying expertise, being part of the emergency services fostered ‘mutual recognition and respect’ due to shared values. Another participant emphasised similar reasons for joining their respective services, promoting a shared frame of reference, helping them see each other as part of the same team:

We are pretty much all of a same mindset. Everyone joins their respective services to to to be that hero and to make that difference, we just do it in slightly different ways. But essentially erm if you stripped us of our […] colours, you probably won’t be able to tell the difference in the service […] so yeah, straight away you’ve got that […] shared sort of frame of reference to each other. (Police, Group 2)

#### Effect of shared identity on joint working

This section is separated into three themes: motivation to work with each other, confidence, and increased trust and respect.

*Motivation to work with each other.* Over half of the groups discussed that forming bonds during the exercise motivated collaboration, for example through listening to each other’s input. Two groups mentioned organisational differences that made working together challenging, for example, different appetites to risk (e.g. Group 2). However, despite these differences, participants acknowledged that established relationships prevented these differences from hindering group working:

There will always be a pull from each other’s own services […] it is the relationships that build around that will stop those conversations from happening. (FRS, Group 2)

Some groups emphasised the importance of clear communication to ensure mutual understanding between participants. For example, in Group 5 one participant said they were actively considering how to communicate with participants from other organisations:

That’s your point of view, well how can I put across my point of view and my understanding to you in your terms or something’s that’s gonna make a lot more sense for you. (Police)

In addition, some groups also discussed wanting to seek information from other organisations before making decisions, especially on topics which were outside of their expertise. For example, Group 4 discussed ‘referring and deferring’ to each other’s expertise rather than making decisions independently and this was driven by the relationships established early in the exercise.

*Confidence.* Several groups highlighted that building bonds fostered participants’ confidence in both others’ ability and their own. In Group 2 a participant said that forming relationships enabled them to communicate more technically because they had confidence the other participants would understand.

Additionally, an established bond between participants fostered confidence in challenging each other when needed. Participants in Group 1 discussed how during the shooting scenario, the Ambulance participant challenged a decision by the Police to not allow them to enter the scene. When asked why they challenged this, the Ambulance participant said that they felt comfortable doing so due to the established working relationship.

An established bond between participants not only gave participants confidence to challenge each other but also confidence when challenged. In Group 6 an FRS participant said they felt comfortable responding to a challenge from a Police participant regarding the risk posed by three feet of water. They described a supportive atmosphere enabling open exchanges due to the relationships between them.

*Increased trust and respect.* Half the groups discussed the importance of trusting each other during the response. In Group 2, trust was established early on via introductions where participants stated their identities and roles.

Establishing trust through understanding each other’s roles was particularly important, especially when scenarios fell outside of participants’ expertise. For example, in Group 2, one FRS participant said they were ‘heavily relying’ on the Police participant to lead due to the scenario not being within their area of expertise. Yet, participants said communicating ‘often and clearly’ with the other participants in the group enabled trust in other participants to develop through recognising ‘everyone brings something to the table’.

Participants said that trust also stemmed from the bond formed between participants through shared principles, like JESIP. In Group 2, JESIP was described as a ‘golden thread’ that guided discussions. Similarly, in Group 4 JESIP served as a common element, fostering ‘mutual recognition and respect’ among participants. This group said this facilitated group working through making them want to get along with each other, unlike past incidents where they might have seen themselves as individual organisations.

## Discussion

The current study explored the relationship between shared identity and interoperability in six multi-agency emergency response groups through discussion-based exercises. It aimed to explore if participants felt a shared identity, how they worked together, and if shared identity was associated with interoperability.

This study found a positive relationship between participants’ identity and their perceived joint working performance, indicating an important role of shared identity in interoperability. Interestingly, although participants felt a sense of shared identity before the exercise discussions began, it was not directly linked to their perceived joint working performance. This suggests two important findings. First, shared identity can develop as a result of spending time together, for example through working together in the exercises, or through being part of the same study. Second, increased shared identity is associated with improved self-perceived joint working performance. This expands on previous research, showing that not only can the time spent together facilitate a sense of shared identity (e.g. Davidson *et al*. 2022), but also that this developed identity is related to improved joint working, thus providing an important advance in our understanding of the role of shared identity in interoperability within emergency response multiteam systems (MTSs).

An earlier study demonstrated that identifying with the overarching MTS improved team performance (Cuijpers *et al*. 2016). However, they focused on undergraduate students, limiting its applicability to real-world responders. Thus, our study marks the first crucial investigation into shared identity’s role in interoperability with this specific population of interest.

In our study, shared identity was associated with improved joint working, evident in the focus group discussions where it fostered motivation, confidence, trust, and respect among responders. This aligns with existing research demonstrating how shared identity improves group dynamics, including increased trust, respect, and cooperation between group members (Reicher *et al*. 2010, Neville and Reicher 2011). In addition, shared identity has been shown to improve coaction between group members (Drury and Reicher 2020). Further research has shown that MTSs coordinate more effectively when their sub-groups share an identity (Cuijpers *et al*. 2016, Mell *et al*. 2020). Thus, our study highlights the important impact of shared identity on group behaviour, particularly within emergency response MTSs.

Shared identity impacts not only multi-agency response teams but also various emergency services that collaborate with other organisations. In policing, the concept of ‘plural policing’ is used to demonstrate the frequent need for collaborative work with other organisations (Crawford *et al*. 2005). For example, child safeguarding entails inter-organisational collaboration among Police, healthcare, education, social care, and youth services (Crawford and L’Hoiry 2017). Effective intergroup working in such contexts relies on factors like shared commitment, purpose, and trust, as noted by Crawford and L’Hoiry (2017) and supported by other studies on plural policing (Whelan 2017, Nøkleberg 2020).

As expected, in our study participants with more prior knowledge of JESIP reported better joint working during the exercise, aligning with the standardised guidance it offers for interoperability. However, despite its established principles challenges persist in implementing JESIP effectively in real-world responses, as documented in emergency response literature (e.g. Pollock 2013) and incident inquiries (e.g. Saunders 2022a).

Upon further exploration, whilst the questionnaire data suggested participants perceived effective joint working, analysis of discussion transcripts revealed challenges. Instances of ‘silo thinking’ emerged, where participants disregarded certain problems as others’ concerns and assumed tasks would be handled by other organisations. This indicates a focus on individual sub-goals over the overarching shared goal, potentially neglecting the needs and priorities of other participants. This aligns with Cuijpers *et al*.’s (2016) findings on inter-team conflicts impacting MTS performance. The qualitative findings in our study support this, illustrating how potential conflicts between sub-groups could affect joint working (e.g. through differing expectations of roles).

Previous research has highlighted the role of pre-existing relationships in fostering interoperability among emergency responders (Davidson *et al*. 2023). In this study, most participants reported having such relationships. Davidson *et al*. (2023) found that these relationships, coupled with recent shared experiences, facilitated group working during crises like the COVID-19 pandemic. In our study, despite pre-existing relationships, participants’ shared identity increased during the exercise which was associated with improved perceived joint working. This suggests that while pre-existing relationships aid present group dynamics, joint working can further strengthen these bonds.

Though our study focused on UK emergency response, its implications extend internationally. Research from various countries, such as the Netherlands (Bharosa *et al*. 2010), Australia (Cattermole-Terzic and Horberry 2020), and Norway (Eide *et al*. 2014), along with inquiries into incidents like the 2010 Haiti earthquake (Arnaouti *et al*. 2022) and Hurricane Katrine in California, USA (Comfort and Haase 2006) reveal similar challenges. For example, Eide *et al*. (2014) found communication gaps among emergency responders in Norway, including issues with shared language and situational awareness. While further research is needed to validate the direct applicability of our findings to other countries’ emergency response teams, the shared challenges suggest relevance, emphasising the role of shared identity in addressing such issues.

### Strengths, limitations, and recommendations for future research

Our study builds on previous research by exploring the link between shared identity and interoperability. Its mixed-methods approach offers a comprehensive understanding of the role of shared identity in interoperability. But there are also limitations that need to be addressed. First, there were unequal participant numbers and representation from emergency services across groups, potentially affecting group dynamics. Whilst findings were consistent across groups, inclusion of all services would enhance generalisability. Future research should therefore consider representation across services.

Second, the study faced challenges with low sample size due to difficulty in recruiting emergency responders because of busy work schedules and operational commitments. It is a well-documented problem that low samples are a common challenge in MTS research (e.g. Alison *et al*. 2015, Bell *et al*. 2018, Brown *et al*. 2021, Wilkinson *et al*. 2019). However, conducting research specifically with the population of interest is essential within this research area to ensure findings are relevant to the population to whom the research is aimed at (Boulton and Cole 2016). Despite sensitivity analyses addressing power, caution is needed in interpreting resulting, particularly in correlational analysis. In addition, group-level analysis may be more appropriate due a potential concern of non-independent samples as participants took part in the study in groups, however, small group sizes limit this approach. Whilst further research is warranted, this study provides valuable insights into shared identity and interoperability.

Third, the study lacked experimental manipulation and therefore whilst relationships between variables can be explored, causal effects cannot be determined. Discussion-based exercises, whilst informative, lack the complexities and pressures of a real incident due to the artificial environment. In the current study this was exacerbated by the virtual nature of the teams. Future research should consider experimental design to allow any causal relationships to be explored, as well as considering high-fidelity environments such as large-scale exercises (e.g. Waring *et al*. 2020) or immersive simulations (e.g. Wilkinson *et al*. 2022) to better understand shared identity’s role in interoperability.

Whilst this paper focused on the relationship between shared identity and interoperability, other factors like governance (e.g. Lakoma 2023) and technology (e.g. Bharosa *et al*. 2010, Abdeen *et al*. 2021) can also influence multi-agency response effectiveness. Therefore, recommendations from this research should be considered alongside these other factors.

Finally, in this study, we used the Joint Doctrine principles (JESIP 2021) as a framework to assess participants’ joint working effectiveness. Whilst this approach aligns with standardised emergency responder training, it lacks validation and may overlook key behavioural and psychological interoperability indicators. To advance interoperability research, future research should develop and validate a systematic framework for interoperability analysis.

### Conclusions

This study is the first, to our knowledge, to provide evidence of a relationship between shared identity and improved group working during multi-agency emergency response. This highlights the important role of psychological factors in fostering interoperability. Our findings highlight how developing a shared frame of reference and emphasising shared goals fosters the development of a shared identity. Based on this, we argue that psychological factors, such as shared identity, should be integrated into multi-agency training and guidance for emergency responders to support preparedness for emergency requiring interoperability.

### Practitioner points

- Shared identity facilitates improved joint working through providing responders with motivation to work with each other, fostering mutual confidence, and increasing levels of trust and respect among team members.
- The development of a shared identity among responders is facilitated when responders align on shared goals and possess a unified framework such as practical principles like JESIP, or shared intrinsic motivations, which foster teamworking.
- Training and guidance for interoperability should prioritise the inclusion of psychological factors, particularly those that foster and highlight a sense of shared identity among responders.

*Ethics statement*: Ethical approval was obtained from the UK Health Security Agency Research and Governance Group, approval number: R&D 477.

## Supplementary Material

Supplementary_Materials_1.docx

Supplemental material

Supplemental material

Supplemental material

Supplemental material

## Data Availability

The data that support the findings of this study are available on request from the corresponding author. The data are not publicly available due to ethical restrictions.
