# Supplementary material for: Exploring the relationship between shared identity and interoperability: a mixed methods analysis of discussion-based multi-agency emergency response exercises
Source: Policing Soc. 2024 Jul 9;35(1):118–34. doi: 10.1080/10439463.2024.2374834 (PMC11649211; doi:10.1080/10439463.2024.2374834)
Supplement: Supplementary_Materials_1.docx [file GPAS_A_2374834_SM3658.docx]

**Supplementary Materials 1**

*Scenarios*

**Scenario 1: Flooding**

It’s Monday 5th December and your local area has just experienced a flash flood. The Environment Agency’s Live Flood Warning Map service had issued a flood alert for the area, but the flash flood was more severe than originally predicted. You have been informed that a local primary school, with 200 pupils, has been severely affected. Some teachers and pupils have evacuated the building to a nearby unaffected Church before the water levels inside the building rose, but others remain inside.

You are an operational commander en route to the scene.

**You have just arrived at the scene. What do you do?**

Inject 1: You and the other commanders have established that the water inside and outside of the school is at a depth of about 3ft. There are 80 children, 6 teachers and 2 support staff still trapped in the building.

**What do you do?**

Inject 2: Initial reports from the Environmental Agency is that further flash flooding is imminent and expected within the next two hours.

**What do you do?**

Inject 3: Everybody has been safely rescued from the school. However, local authorities have said the school is vital for the community and want the school to be protected from further damage.

**What do you do?**

**Scenario 2: Marauding Terrorist Firearms Incident**

At 19:30 on Friday 12th August reports are received of gunshots at Pizza Express on the local High Street. There are thought to be about 40 people inside the restaurant. There are initial reports of several severely injured victims, although the extent of injuries is currently unknown. The location of the gunman is currently unknown. The police have declared Operation Plato.

You are an operational commander en route to a Police designated RVP.

**You have just arrived at the RVP. What do you do?**

Inject 1: The first paramedics deployed into the warm zone have conducted an initial triage and reported there are 41 casualties. Three people have been declared dead, including the suspected gunman. Surviving casualties are reporting there was just one gunman. Walking wounded are beginning to leave the scene and uninjured onlookers are trying to enter the premises to help the injured.

**What do you do?**

Inject 2: Media have arrived at the cordons and are requesting updates on shooting, as well as live streaming the events to media channels. You have heard that there has been speculation on social media that a second gunman was present during the attack and fled the scene. Casualties inside the venue are saying that they only saw one gunman present.

**What do you do?**

Inject 3: Police have intelligence suggesting that this was an isolated incident and that there is no evidence to suggest a second gunman was present on the scene, or that a second attack is likely to take place.

**What do you do?**
