# Supplementary material for: Exploring the relationship between shared identity and interoperability: a mixed methods analysis of discussion-based multi-agency emergency response exercises
Source: Policing Soc. 2024 Jul 9;35(1):118–34. doi: 10.1080/10439463.2024.2374834 (PMC11649211; doi:10.1080/10439463.2024.2374834)
Supplement: Supplemental material [file GPAS_A_2374834_SM3657.docx]

**Supplementary Materials 4**

*Focus group topic guide*

1. Did you feel a bond with the other responders taking part in this exercise?

Prompts:

A) What factors facilitated this?

B) What factors interfered with this?

2. Do you think that the other responders taking part in this exercise felt a bond with each other?

Prompts:

A) What factors facilitated this?

B) What factors interfered with this?

3. How closely do you feel you followed the JESIP guidelines?

Prompts:

A) How well do you think you discussed co-locating with each other?

B) How well do you think you communicated with each other?

C) How well do you think you co-ordinated with each other?

D) Do you think you had a joint understanding of risk?

E) Do you think you had shared situational awareness?

4. Is there anything else that you think it is important to add in relation to the group’s performance of the exercise?
