# Supplementary material for: Exploring the relationship between shared identity and interoperability: a mixed methods analysis of discussion-based multi-agency emergency response exercises
Source: Policing Soc. 2024 Jul 9;35(1):118–34. doi: 10.1080/10439463.2024.2374834 (PMC11649211; doi:10.1080/10439463.2024.2374834)
Supplement: Supplemental material [file GPAS_A_2374834_SM3655.docx]

**Supplementary Materials 2**

*Pre-exercise questionnaire*

On a scale of 1-7, what extent do you agree with the following statements:

· ‘I feel a bond with the other responders taking part in this exercise.’

· ‘I think that the other responders taking part in this exercise feel a bond with each other.’

| 1 | 2 | 3 | 4 | 5 | 6 | 7 |
| --- | --- | --- | --- | --- | --- | --- |
| Strongly disagree | Disagree | Somewhat disagree | Neither agree nor disagree | Somewhat agree | Agree | Strongly Agree |
