# Supplementary material for: Exploring the relationship between shared identity and interoperability: a mixed methods analysis of discussion-based multi-agency emergency response exercises
Source: Policing Soc. 2024 Jul 9;35(1):118–34. doi: 10.1080/10439463.2024.2374834 (PMC11649211; doi:10.1080/10439463.2024.2374834)
Supplement: Supplemental material [file GPAS_A_2374834_SM3654.docx]

**Supplementary Materials 5**

*Sensitivity analysis*

We conducted sensitivity analyses for RQs 1-3 using G*Power (Faul et al., 2007) to determine whether there was sufficient power in the analysis to detect effects.

For RQ 1, A one-sample Wilcoxon signed rank test with 24 participants would be sensitive to effects of d = .65 with 80% power (alpha = .05, two-tailed). The results from the one-sample Wilcoxon signed rank tests reached effect sizes (r = .77, .85, .84, respectively) which, converted (d = 2.41, 3.23, 3.10), did not fall below the value of d = .65.

For RQ2, a one-sample Wilcoxon Signed Rank Test with 24 participants would be sensitive to effects of d = .65 with 80% power (alpha = .05, two-tailed). The results from the one-sample Wilcoxon Signed Rank Tests reached effect sizes (r = .87 for both tests) which, converted (d = 3.53), did not fall below the value of d = .65. In addition, a related-samples Wilcoxon Signed Rank Test with 24 participants would also be sensitive to effects of d = .65 with 80% power (alpha = .05, two-tailed). The results from the related-samples Wilcoxon Signed Rank Test reached an effect size (r = .37) which, converted (d = .78), did not fall below the value of d = .65.

For RQ3, a Spearman’s correlation coefficient with 24 participants would be sensitive to the effects of r = .48 with 80% power (alpha = .05, one-tailed). This means the study would not be able to reliably detect correlations smaller than r = .51. This indicates that some correlations in this study were underpowered.

This indicates that our study was partially underpowered.
